# Characterization and phylogenetic analysis of the complete chloroplast genome sequence of *Pertya multiflora* (Asteraceae), a rare and endangered wild plant species in China

**DOI:** 10.1080/23802359.2021.1936671

**Published:** 2021-06-07

**Authors:** Ye Liu, Huijuan Zhang, Qian Wu, Wenxin Huang, Tao Wu, Ming Guan, Ming Jiang

**Affiliations:** College of Life Sciences, Taizhou University, Jiaojiang, China

**Keywords:** *Pertya multiflora*, chloroplast genome, phylogenetic analysis, rare plant species

## Abstract

*Pertya multiflora* (Asteraceae) is a rare wild plant species narrowly distributed in Zhejiang province, China. In our present study, we assembled its complete chloroplast genome using high-throughput sequencing data. The results indicated that the whole chloroplast genome of *P. multiflora* was 153,396 bp in length. Its large single copy, small single copy, and inverted region sequences were 84,575 bp, 18,451 bp, and 25,185 bp. The *P. multiflora* chloroplast genome was composed of 134 genes, including 87 protein-coding genes, 37 tRNA genes, eight rRNA genes, and two pseudogenes. Phylogenetic analysis results showed that *P. multiflora* was grouped with *Gerbera jamesonii*, with 100% bootstrap support.

Asteraceae is the largest flowering-plant family found throughout the world except for Antarctica, and it is composed of approximately 1620 genera and 23,600 species (Funk et al. 2005). *Pertya* is a small genus in the family Asteraceae, which consists of approximately 25 plant species. *Pertya* plants are divided into four series, including *Paniculatae*, *Phylicoides*, *Scandentes*, and *Sinenses*. *Pertya* plants are distributed in Asia countries like China, Japan, Thailand, and Afghanistan, but most of them are found in China (Wu et al. 2011). Plants in the genus *Pertya* are shrubs, subshrubs, or perennial herbs, rarely scandent shrubs (Murata and Ohi-Toma 2016). In recent years, the studies on *Pertya* have been focused on phytochemistry, mating system, and taxonomy (Nagai et al. 1975; Ohtsuka et al. 2005; Zhang et al. 2013). Nagai et al. (1975) isolated a new triterpene methyl ether designated as O-methyl pertyol from the roots of *P*. *robusta*, and it was identified as the first C_33_ triterpene methyl ether. Nagumo et al. (1982) isolated a new sesquiterpene dilactone, namely pertilide, from *P*. *glabrescens*, and its structure was determined by X-ray crystallographic analysis. Ohtsuka et al. (2005) estimated genetic diversities and inbreeding coefficients of six Asteraceae plants, including *P. glabrescens* and *P. scandens*, with the highest diversity values observed in the two *Pertya* species. *P. multiflora* Cai F. Zhang & T. G. Gao 2013 is a new plant species distributed only in Zhejiang province, and it has been evaluated as critically endangered using the IUCN red list criteria (Zhang et al. 2013). In our present study, the complete plastid genome of *P. multiflora* was assembled, characterized, and investigated for its phylogenetic position.

Fresh leaves of *P. multiflora* were collected from Shuimokeng valley (28°52.043 N, 121°07.522E), Linhai county, Zhejiang province, China. The leaves were rinsed with distilled water and kept in a −80 °C refrigerator for DNA extraction. A specimen was deposited at the Molecular Biology Laboratory at Taizhou University (Ming Jiang, jiangming1973@139.com) under the voucher number of CHS2016003. Genomic DNA was isolated following the cetyl trimethyl ammonium bromide (CTAB) protocol of Doyle and Doyle (1987). A DNA library was constructed following the standard protocol supplied by Illumina, and it was then sequenced on the Hiseq X Ten sequencing platform at 150 bp paired-end (PE). Approximately 2.98 Gb raw reads were generated, and they were then filtered by NGS QC Toolkit v2.3.3 to yield 2.96 G high-quality clean reads (Patel and Jain 2012). NOVOPlasty, a seed-extend based *de novo* assembler, was used to assemble the chloroplast genome (Dierckxsens et al. 2017). An online program DOGMA (Dual Organellar GenoMe Annotator, http://dogma.ccbb.utexas.edu/) was applied to annotate the chloroplast genome (Wyman et al. 2004).

Overall, we yielded 9,864,304 bp PE clean reads after trimming poor quality bases and filtering low-quality reads, and they were used to produce a circular plastid by NOVOPlasty. The complete chloroplast genome sequence of *P. multiflora* is 153,396 bp in length with an LSC region (84,575 bp), an SSC region (18,451 bp), and two copies of IRs (25,185 bp). Annotation results demonstrated that the *P. multiflora* chloroplast genome harbored 134 genes, including 87 protein-coding genes, 37 tRNA genes, eight rRNA genes, and two pseudogenes. Among these genes, six protein-coding genes (*ycf2*, *ndhB*, *ycf15*, *rps7*, *rpl23*, and *rpl2*), eight tRNA genes (*trnG-UCC*, *trnN-GUU*, *trnR-ACG*, *trnA-UGC*, *trnI-GAU*, *trnV-GAC*, *trnL-CAA*, *trnI-CAU*), and four rRNA genes (*23S* rRNA, *5S* rRNA, *4.5S* rRNA, and *16S* rRNA) contain two copies. The 3′ ends of *ycf1* and *rps19*, locating at 3′ and 5′ ends of IRa respectively, were found to be truncated and thus formed two pseudogenes. We have submitted the annotated chloroplast genomic sequence to GenBank under an accession number of MW148616.

The complete chloroplast genome sequences of five *Ligularia* species (MF539932, MN783367, NC_039383, NC_039382, and KT988070), five *Cynara* (KP299292, KP842705, KP842706, KP842707, KP842715), five *Artemisia* (MG951482, MG951484, MG951490, MG951492, MG951496), four *Saussurea* (NC_044727, NC_044732, NC_044734, NC_044739), four *Echinacea* (KX548219, KX548222, KX548224, KX548223), six *Atractylodes* (NC_044671, NC_037484, NC_037483 MW301113, MT834523, MT834524), and three other Asteraceae plants together with *P. multiflora* and the outgroup *Adenophora stricta* (NC_036223) were aligned to construct a phylogenetic tree. The sequences were aligned using MAFFT v7.388 (Katoh and Standley 2013), and the best-fitting nucleotide substitution model for maximum likelihood (ML) analysis was selected by running jModelTest (Darriba et al. 2012). GTR + G + I was turned out to be the best DNA substitution model. A maximum likelihood (ML) tree was then generated using PhyML 3.1 (Guindon et al. 2010). Phylogenetic analysis results were well matched to taxonomic classification, and plant species within the same genus clustered on the same clade. Our results indicated that *P. multiflora* grouped with seven plants from genera *G jamesonii*, with 100% bootstrap support (Figure 1).

Characterization and phylogenetic analysis of the plastid sequence of *P. multiflora* will provide insights into genetic diversity studies and conservation of this rare plant species in the future.

## Data Availability

The data that support the findings of this study are openly available in GenBank of NCBI at https://www.ncbi.nlm.nih.gov/nuccore/MW148616. The associated BioProject, SRA, and Bio-Sample numbers are PRJNA688695, SRR13329723, and SAMN17185260, respectively. A maximum likelihood tree based on the complete chloroplast genome sequences of *Pertya multiflora* (Asteraceae) and related species, with *Adenophora stricta* as the outgroup. The numbers next to nodes indicate bootstrap support values.
